# Long term outcomes and causal modelling of compulsory inpatient and outpatient mental health care using Norwegian registry data: Protocol for a controversies in psychiatry research project

**DOI:** 10.1002/mpr.1980

**Published:** 2023-07-08

**Authors:** Tore Hofstad, Olav Nyttingnes, Simen Markussen, Erik Johnsen, Eoin Killackey, David McDaid, Miles Rinaldi, Kimberlie Dean, Beate Brinchmann, Kevin Douglas, Linda Gröning, Stål Bjørkly, Tom Palmstierna, Maria Fagerbakke Strømme, Anne Blindheim, Jorun Rugkåsa, Bjørn Morten Hofmann, Reidar Pedersen, Tarjei Widding‐Havneraas, Knut Rypdal, Arnstein Mykletun

**Affiliations:** ^1^ Centre for Research and Education in Forensic Psychiatry Haukeland University Hospital Bergen Norway; ^2^ Centre for Medical Ethics University of Oslo Oslo Norway; ^3^ Health Services Research Unit Akershus University Hospital Lørenskog Norway; ^4^ Ragnar Frisch Centre for Economic Research Oslo Norway; ^5^ Division of Psychiatry Haukeland University Hospital Bergen Norway; ^6^ Department of Clinical Medicine University of Bergen Bergen Norway; ^7^ NORMENT Centre of Excellence Haukeland University Hospital Bergen Norway; ^8^ Orygen Melbourne Australia; ^9^ Centre for Youth Mental Health The University of Melbourne Melbourne Australia; ^10^ Care Policy and Evaluation Centre Department of Health Policy London School of Economics and Political Science London UK; ^11^ Centre for Work and Mental Health Nordland Hospital Trust Bodø Norway; ^12^ South West London and St George's Mental Health NHS Trust London UK; ^13^ Discipline of Psychiatry and Mental Health School of Clinical Medicine University of New South Wales Sydney Australia; ^14^ Justice Health and Forensic Mental Health Network Sydney NSW Australia; ^15^ Department of Psychology Simon Fraser University Vancouver British Columbia Canada; ^16^ Regional Centre for Research and Education in Forensic Psychiatry Oslo University Hospital Oslo Norway; ^17^ Faculty of Law University of Bergen Bergen Norway; ^18^ Faculty of Health and Social Sciences Molde University College Molde Norway; ^19^ Department of Clinical Neuroscience Centre for Psychiatric Research Karolinska Institutet Stockholm Sweden; ^20^ Faculty of Medicine and Health Sciences Department of Mental Health Norwegian University of Science and Technology (NTNU) Trondheim Norway; ^21^ Centre for Care Research University of South‐Eastern Norway Porsgrunn Norway; ^22^ Department of Mental Health Oslo Metropolitan University Oslo Norway; ^23^ Faculty of Medicine and Health Sciences Department of Health Sciences Norwegian University of Science and Technology Gjøvik Norway; ^24^ UiT—The Arctic University of Norway Tromsø Norway; ^25^ Division for Health Services Norwegian Institute of Public Health Oslo Norway

**Keywords:** causal inference, coercion, compulsion, compulsory mental health care, geographical variation, instrumental variables, psychiatric epidemiology

## Abstract

**Objectives:**

Compulsory mental health care includes compulsory hospitalisation and outpatient commitment with medication treatment without consent. Uncertain evidence of the effects of compulsory care contributes to large geographical variations and a controversy on its use. Some argue that compulsion can rarely be justified and should be reduced to an absolute minimum, while others claim compulsion can more frequently be justified. The limited evidence base has contributed to variations in care that raise issues about the quality/appropriateness of care as well as ethical concerns. To address the question whether compulsory mental health care results in superior, worse or equivalent outcomes for patients, this project will utilise registry‐based longitudinal data to examine the effect of compulsory inpatient and outpatient care on multiple outcomes, including suicide and overall mortality; emergency care/injuries; crime and victimisation; and participation in the labour force and welfare dependency.

**Methods:**

By using the natural variation in health providers' preference for compulsory care as a source of quasi‐randomisation we will estimate causal effects of compulsory care on short‐ and long‐term trajectories.

**Conclusions:**

This project will provide valuable insights for service providers and policy makers in facilitating high quality clinical care pathways for a high risk population group.

**Abbreviations**
ADHD: Attention Deficit Hyperactivity Disorder
CPR: The Central Population Register
CRPD: Convention on the Rights of Persons with Disabilities
IV: Instrumental Variable
NPR: The Norwegian Patient Register
OC: Outpatient Commitment
RCT: Randomised Controlled Trial
SMI: Severe Mental Illness
WP: Work Package

## 1 INTRODUCTION

In all mental health care systems, there are limits on what is known about beneficial outcomes of different care pathways; but clinical decisions must nevertheless be made, leading to practice variations. Since this variation is typically unknown to service users, it can enable quasi‐experimental designs, valuable in a context in which Randomised Controlled Trials (RCT) are difficult, or even impossible, to conduct. This protocol is part of the project “Controversies in Psychiatry” (Mykletun et al., 2021), whose main aim is to use clinical practice variation observed in Norwegian national registries to obtain knowledge on key questions that for practical, legal, and ethical reasons are hard to answer through RCTs. The first controversy project investigated Attention Deficit Hyperactivity Disorder (ADHD) (Mykletun et al., 2021); this project highlights controversies surrounding compulsory inpatient and outpatient mental health care, including administration of medication without consent.

Insufficient high‐quality evidence on the efficacy and effectiveness of healthcare pathways creates clinical uncertainty about trade‐offs between benefits and harms for patients, and for the healthcare system and larger society. This is one reason why controversies are common in mental health care. One such controversy concerns compulsory care in hospital and community settings. Many patients experience compulsion as harmful (Nyttingnes, 2018), and report few benefits (Katsakou and Priebe, 2007). Governments (Sosial‐og Helsedirektoratet, 2006), user organisations (The National Coalition of Mental, 2015), and professionals, including the World Psychiatric Association (Herrman et al., 2022), have requested a reduction of compulsory mental health care. There are also calls for alternatives to coercion (Gooding, 2021), emphasising co‐developed options, supported decision‐making and increased user‐participation, particularly following the United Nations' Convention on the Rights of Persons with Disabilities (CRPD) (UN General Assembly, 2007). Meanwhile, mental health services are often criticised for failing to protect citizens and for not using sufficient coercion, following what remain rare acts of violence by people with severe mental illnesses (SMI) (Honningsøy & Radøy,  2021), as well as post‐discharge suicide (Moland & Zondag,  2014).

In Norway, psychiatric inpatient bed numbers have been reduced over decades with more mental health service users receiving community‐based support (Lilleeng et al., 2014), a development also seen internationally (World Health Organization, 2022). This includes compulsory community services, such as Outpatient Commitment (OC), which uses legal instruments to compel people to receive outpatient services.

### 1.1 The purpose of compulsory care

The purpose of compulsory mental health care is to reduce the short‐term risk of suicide or self‐harm and reduce the risk that the person will be a victim or perpetrator of violence or acts that violate others' life, health, or freedom. In the long term, mental health care, including compulsion, is expected to facilitate recovery and prevent relapse. Improved outcomes can potentially impact beyond health and risks of harm, to influence educational attainment; employment and welfare reliance; relationships and childcare; and criminal justice involvement.

The relationship between inpatient and outpatient compulsory care remains unclear. As both are imposed on patients without their consent, it may be worthwhile to examine these practices together.

## 2 RISK OF VIOLENCE AMONG PERSONS WITH SMI

When violent acts are committed by individuals with a history of mental illness, the media often highlights these incidents, leading to public debates that often blame clinicians and the healthcare system for failing to provide compulsory mental health care. These discussions generally overlook the complexity involved in deciding when to use compulsory mental health care and make assumptions about its effectiveness in preventing such incidents. The risk of violence (Dack et al., 2013) or suicide (Inskip et al., 1998) among those with SMI remains low, making risk assessment difficult, as well as producing false positives who may suffer unnecessarily from the detrimental impacts of compulsory care (Palmstierna, 1999; Rosen, 1954). Moreover, individuals with SMI are more likely to be victims of violent crime than perpetrators (Latalova et al., 2014).

### 2.1 Existing evidence for effectiveness of inpatient and outpatient compulsory care

Norwegian legislation enabling compulsory mental health care (Psykisk helsevernloven med kommentarer, 2022) presupposes that compulsory care is effective, by using words such as *necessary*, which implies that the situation is expected to improve from compulsory care, compared to the counterfactual scenario without it. There is, however, limited evidence to support this assumption in relation to many outcomes, including the prevention of suicide (Zalsman et al., 2016). A recent study from Norway found no worse outcomes for people with SMI residing in areas with little use of compulsory mental health care, compared to areas with frequent use (Nyttingnes et al., 2023). A Cochrane review (Kisely et al., 2017) identified three RCTs on OC, one in England (Burns et al., 2013) and two in the United States (Steadman et al., 2001; Swartz et al., 2001). They all failed to find a difference between OC and standard “voluntary” care in relation to service use, social functioning, mental state, or life quality. OC recipients were, however, less likely to be crime victims. The Cochrane review concluded these trials were of low to medium quality and called for higher quality research. Longer term analysis of the English OC trial indicated no overall cost difference between the OC group and a voluntary treatment comparator group even from a societal perspective, with no difference in health or capability‐related life quality (Simon et al., 2021). Long term follow up similarly found no association between OC and improved social functioning 4 years later (Vergunst et al., 2017).

Another review of 60 studies concluded that the evidence is sufficiently strong to infer causally that OC does not prevent relapse or reduce hospital admission (Kisely et al., 2007). In contrast, a review of 39 studies (Segal, 2022) argues OC is associated with reduced all‐cause‐mortality. Beyond the few RCTs implemented, the observational studies conducted to date are susceptible to bias and confounding. Importantly, no RCTs have investigated the effectiveness of compulsory hospitalisation.

### 2.2 The Controversy of Compulsory Care's Effectiveness

The controversy which this project protocol addresses is therefore, first, whether compulsory care^1^ results in reduced levels of mortality, suicides, and serious crime; and secondly, whether compulsory care has positive effects on outcomes such as self‐harm episodes, injuries, unemployment, and welfare reliance among people at high risk of compulsory care.

### 2.3 Why RCTs are not likely to resolve the controversy

Conducting RCTs to investigate the effectiveness of compulsory care is challenging due to practical, legal, and ethical considerations. Double‐blinding care status is impossible, leading to bias risks. Strongly opinionated clinicians can hinder clinical equipoise. For financial and practical reasons, RCTs require a short time frame, making them unsuitable for answering questions related to long‐term outcomes over years. Even if these practical constraints could be mitigated, many ethics committee would not approve randomising people who meet legal criteria for compulsion to voluntary status; the English RCT on OC was required to provide a detailed legal opinion prior to approval (Dawson et al., 2011).

Some commentators argue that RCTs may be suboptimal for evaluating the effectiveness of complex interventions such as OC, which operate in open systems involving social actors (Duncan et al., 2020; Mustafa, 2017; O’Reilly and Vingilis, 2018).

Consequently, it remains unclear whether compulsory mental health care is beneficial, either at the individual or societal level, and whether outcomes expected by legislators are actually delivered. This uncertainty, coupled with differing clinician opinions, provides room for considerable practice variation.

### 2.4 Variations in clinical judgement

**FIGURE 1:**
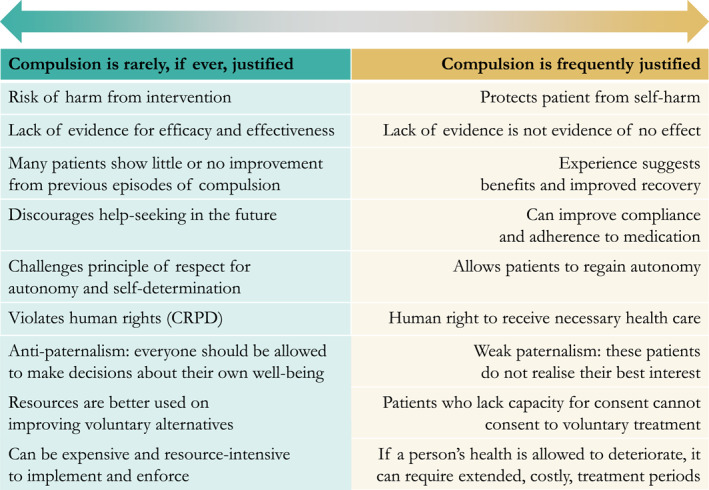
Clinician preference for or against compulsion.

As noted, debate has been ongoing for decades regarding compulsory mental health care. While some argue for reduction (Cratsley et al., 2021; Gooding, 2021), and even abolition of coercion in mental health care (Council of Europe Committee on Social Affairs, 2019; Zinkler and vonPeter, 2019), others believe the focus on autonomy and self‐determination has gone too far, risking inadequate care for patients and negative impacts on their well‐being, and increase risk of harm to others (Freeman et al., 2015). This has led to a spectrum of opinions among clinicians (Aasland et al., 2018; Husum et al., 2017), ranging from those who believe that compulsion can rarely, if ever, be justified to those who believe it can be justified in a substantial proportion of clinical contexts. Figure 1 illustrates this continuum of clinical views, underpinned by arguments to support each pole.

**FIGURE 2:**
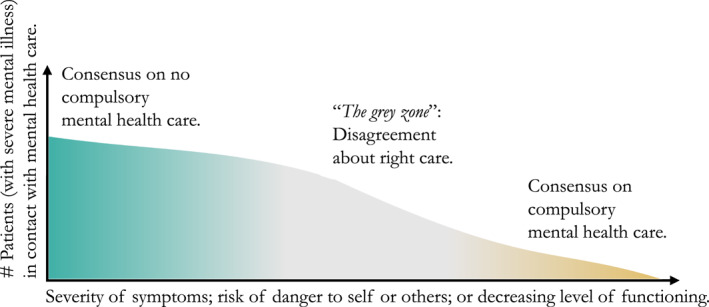
Theoretical illustration of the controversy on the use of compulsory mental health care for people with severe mental illness.

In clinical practice, there is likely to be greater agreement among clinicians that patients with mild symptoms and low risk should not be involuntarily admitted or subject to OC, while those with severe symptoms and high risks may require compulsory treatment (Figure 2). Consequently, the controversy primarily surrounds patients whose symptom levels and risks fall between these two extremes, who are most likely to experience variations in clinical practice dependent on clinician and system preferences. People in this *‘grey zone’* are thus more likely to be managed differently by different clinicians, or in different health systems, producing considerable variation in levels of compulsory care.

### 2.5 Geographical variation in compulsory mental health care: A source of concern and a toolbox for causal inference

Geographical variation in health services is more apparent in conditions where clinicians disagree about *‘right care’* (Wennberg, 2010). It is therefore unsurprising that geographical variation in compulsory hospitalisation is observed in all countries with available data: Belgium (Gedwongen verblijven in PZ en, 2021); Denmark (Engberg, 1991); England (Keown et al., 2016); Finland (The Finnish Institute for Health and Welfare, 2022); France (Gandré et al., 2018); Germany (Brieger et al., 2014); Ireland (Daly and Craig, 2021); Italy (Di Cesare et al., 2017); New Zealand (O’Brien et al., 2012); The Netherlands (Broer et al., 2020); South Korea (Hwang et al., 2020); Sweden (Statistikdatabaser, 2021); Switzerland (Schuler et al., 2018); and the United States of America (Lee and Cohen, 2021). Mental health services are expected to be of similar quality within most of these countries, and quality of care is not presumed to depend on area of residence.

**FIGURE 3:**
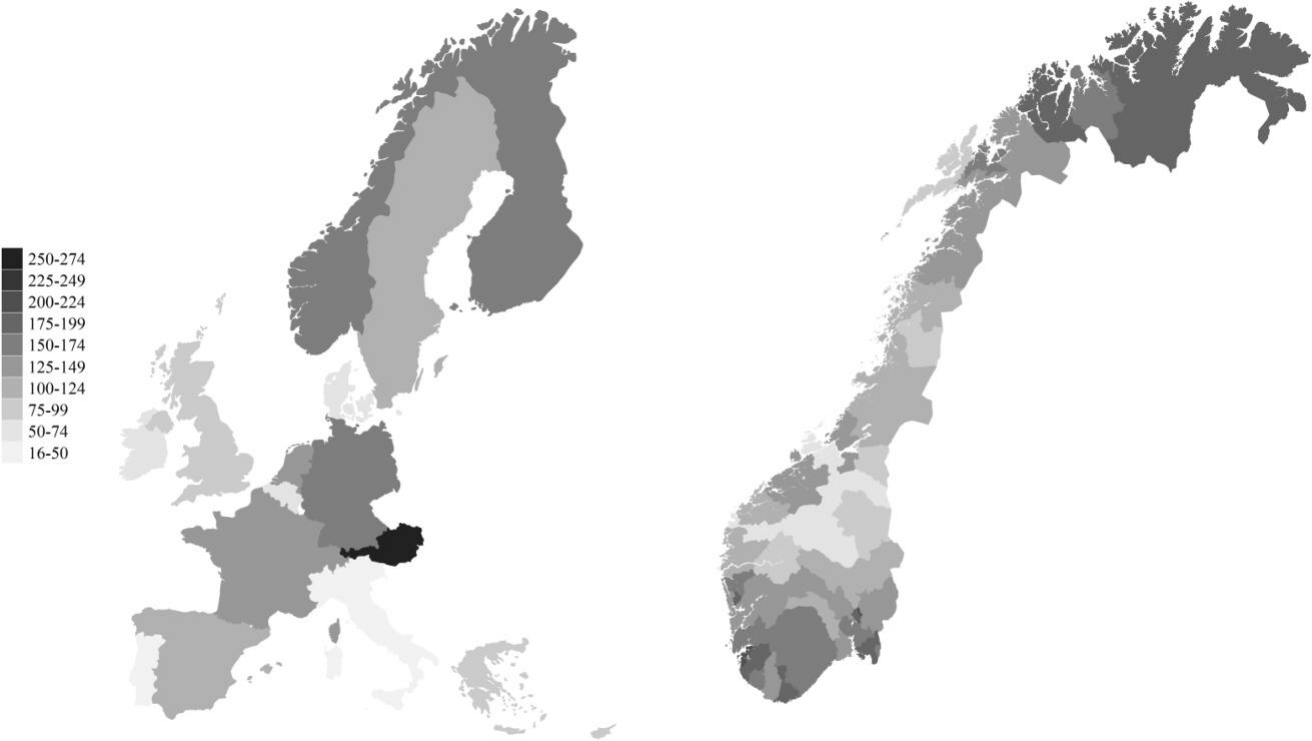
Geographical variation in compulsory hospitalised patients in Europe and Norway. Average rates per 100.000 inhabitant. Map based on data from (Hofstad et al., 2021; Sheridan Rains et al., 2019).

Norway has a universal health care system where all residents are entitled to equal access to mental health care, and there is no separate private mental health sector. Still, after controlling for age and gender of residents, the average number of compulsorily hospitalised patients per person in the population in 2014–2018 was more than three times higher in the highest‐ranked area compared to the lowest (Hofstad et al., 2021), as illustrated on the right panel of Figure 3. Measured as the average number of days of compulsory hospitalisation per capita, the variation was more than eightfold. Compared to other European countries (Sheridan Rains et al., 2019), the average rate of compulsorily hospitalised patients in Norway is relatively high, as seen in the left panel of Figure 3.

There are likely many factors related to the services and their patient populations, that influence the varying rates of compulsory admission. Characteristics of service providers might include bed capacity and duration of hospitalisations (Bale et al., 2021); clinicians' evaluation of decision‐making capacity (Høyer et al., 2022); different attitudes to coercion, which has been shown to vary between professions (Aasland et al., 2018); varying interpretations of legal criteria (Feiring and Ugstad, 2014); availability of systematic family involvement and support for people with SMI (Hestmark et al., 2021); other local experience‐based practises, including quality of voluntary care, which can impact on whether compulsory care is considered necessary (Sharpe, 1997); or other individual differences in clinical decision‐making (Bagby et al., 1991). Variation related to case‐mix might include differing characteristics among the population‐at‐risk of compulsory care, such as age, gender, ethnicity, employment status, comorbidity and previous history of compulsion (Barnett et al., 2019; Walker et al., 2019).

### 2.6 The geographical lottery

For people with SMI, the geographical variation produces a lottery‐like situation. In one area, specialists might follow an early compulsory care approach, based on the expectation that admission and/or pharmaceutical treatment is in their best interest, whether they agree or not. In the neighbouring area, specialists may believe that patient autonomy and long‐term therapeutic relationship are more important and are inclined to wait and see if a crisis can be averted or less restrictive interventions can be provided voluntarily.

The care that patients receive based on such idiosyncratic decisions can appear arbitrary and mimics participating in an RCT. Geographical variation in compulsory mental health care thereby enables knowledge production by providing a quasi‐experimental framework which permits causal inference to be drawn from observational registry data. Service providers' preferences can be used as an Instrumental Variable (IV) to estimate the causal effect of a ‘treatment’, such as compulsory care, on various outcomes (Brookhart and Schneeweiss, 2007; Widding‐Havneraas et al., 2021). These data are already being collected in mandatory national registers and cover entire populations, yielding results of high external validity at a low cost.

### 2.7 Provider preference based instrumental variables as alternative to RCT

Unmeasured confounding occurs when unobserved factors affect both treatment and outcome. This can overestimate or underestimate treatment effects. Preference‐based IVs can address this problem by exploiting random variation induced by an instrument to isolate exogenous variation and remove confounding variation, resulting in a quasi‐experimental approach that only utilizes exogenous variation to estimate the treatment effect (Angrist et al., 1996; Widding‐Havneraas et al., 2021). Instrumental variable analyses have been shown to produce estimates comparable to those from randomised experiments (Cook et al., 2008; McClellan et al., 1994; Stukel et al., 2007), in contrast to conventional observational studies (Cook et al., 2008).

The use of IV and registry data appears to be a promising way to estimate causal effects of compulsory care, while circumventing the many obstacles that may prevent RCTs and threaten to bias conventional multivariable regression approaches. The data are collected in a naturalistic context and the results will be representative and relevant for clinical practice. Since we can follow people over years, we can also track long term consequences of compulsory care. To the best of our knowledge, this project will be the first to apply this method to help resolve the controversy on the effectiveness of compulsory mental health care (Widding‐Havneraas et al., 2021) and can thereby push the research frontier on a topic of considerable societal importance.

## 3 OBJECTIVES

**FIGURE 4:**
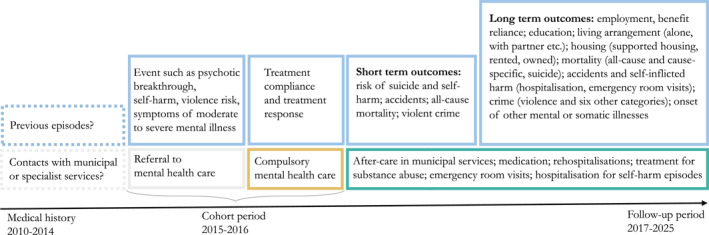
Trajectories observed in longitudinal registry data to be used in the project.

**FIGURE 5:**
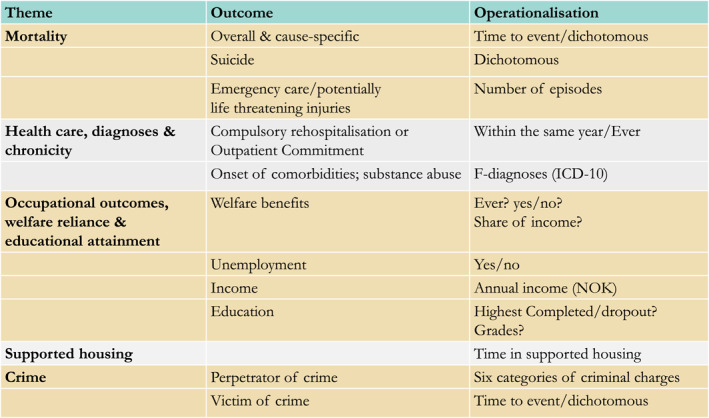
Themes of outcomes and/or baseline characteristics, including potential operationalisation.

The overarching aim is to determine whether compulsory mental health care results in superior, worse or equivalent health and other outcomes for people with SMI. Norwegian registry data on real‐world practice will provide outcomes and trajectories following compulsory care including emergency care/injuries and cause‐specific mortality; comorbidities and substance abuse; compulsory rehospitalisation or outpatient commitment; labour force participation, welfare reliance and educational attainment; supported housing; and serious violence and victimisation, see Figures 4 and 5. The proposed project is organised in two work packages (WP).

### 3.1 WP1: The epidemiology of severe mental illness, compulsory hospitalisation, and outpatient commitment

This WP will answer three categories of epidemiological questions concerning people in contact with specialist mental health or addiction services in Norway in 2015–2016.

The first category will compare short and long‐term trajectories of persons with and without SMI in voluntary and compulsory care. Additionally, the outcomes of these persons will be compared to a control group from the general population.

The second category will describe the extent of geographical variation in

1. Prevalence of SMI.
2. All mental health service use for people with SMI, including referrals to specialist mental health care and per‐person use of antipsychotic medication.
3. Compulsory inpatient and outpatient health service use.

We will test the competing hypotheses that providers who are less likely to use one form of compulsion may be more likely to use the other, or that providers who use little inpatient compulsion also tend to use little OC.

Data will be aggregated to geographical regions, specifically catchment areas to community mental health centres, based on residency. The variation will be described as population‐based rates and as the proportion of inpatients and outpatients with a compulsory referral or legal status. The relationship between urbanicity and risk of psychosis (Fett et al., 2019) has not previously been documented in Norway, but will also be covered in WP1.

The third category will explore associations between rates of compulsory care per population and indicators of social inequality and socio‐economic position in the catchment areas, such as the proportion of high school dropouts; median income in the area; and unemployment rate.

### 3.2 WP2: Short and long‐term causal effects of compulsory inpatient and outpatient mental health care, including administration of medication without consent

The main objective of WP2 is to estimate the causal effect of compulsory mental health care on health and social functioning outcomes for people with SMI, potentially including long term costs to the public purse and society associated with these differences. We will use variation in clinical practice among catchment areas as IV to estimate the causal effect of compulsory care on multiple outcomes (see Figure 5). The primary analyses will be restricted to (a) people with SMI; and (b) people who are referred to compulsory inpatient mental health care.

We expect that patients on the margin of compulsory care after discharge will be treated differently depending on whether they live in areas with higher or lower tendency to use OC. Since OC is most often used to ensure medication compliance (Riley et al., 2019) and almost all people on OC receive antipsychotic treatment (Barkhuizen et al., 2020; Riley et al., 2019; Rugkåsa et al., 2015), we will use provider preference for use of OC as a proxy to measure the effect of administration of medication without consent. This WP will also test assumptions that must be fulfiled for IV‐estimates to credibly reflect causal relationships (Widding‐Havneraas et al., 2021).

## 4 METHODS AND ANALYSIS

### 4.1 Design

The project will use methods of description and causal inference (Hernán et al., 2019). Individual‐level data on the whole Norwegian population will be linked across multiple registries. If uncertainty remains whether conditions for IV‐analyses are met, the use of hierarchical models or inverse probability weighting might be relevant, in addition to conventional epidemiological approaches.

### 4.2 Co‐creation and user involvement

Service users, including people with lived experience of compulsion, have been consulted intermittently during the project planning. We will host seminars with service users and providers to ensure that our research questions are relevant for the users and the clinical field.

### 4.3 Lived experience commentary by Anne Blindheim

As an expert by experience, working as peer support at mental health institutions, I have encountered many persons who suffered from psychosis, each with unique backgrounds and life goals. In discussions with the authors, I expressed disagreement with the term « service user». Individuals like myself, who have undergone compulsory treatment, did not willingly « use the service » but rather had it imposed upon us. Personally, I see myself as a *patient* of psychiatry, getting help and support for my mental health issues, similar to how I would seek medical help for physical conditions such as high blood pressure. But today I am also an employee and a former humanities student. I therefore suggested referring to the individuals in the study within a mental health context as « patients», and using terms such as « person», «employee » or « student » when discussing them in other settings.

One of the planned outcomes concerns employment. While mental illness can be seen as a disease, it can also be seen as a form of social deviation, leading to the rejection of many individuals by society. When someone has experienced marginalisation over time, recovery can result in other positive results than work or education. In my case, I felt rejected by society because of my illness and forced into the fringes. As a result, there may be a sense of ambivalence towards becoming «a good citizen». I therefore asked the researchers to bear in mind that a good outcome is not necessarily to become well adjusted to society, for example, being an employee or a student. It is important to acknowledge that we cannot see the whole picture with this dataset. Another point is that it is not easy to have lived a very different life from that of our colleagues and then attempt to fit in, especially when carrying the stigma associated with SMI. You may have successfully navigated work situations, just not with the burden of that stigma. Thus, the issue may not lie with an individual's ability to work but rather with society's ability to accommodate and accept them.

Putting these considerations aside, I think this project is well thought out and I wholeheartedly support it.

### 4.4 The Norwegian legal framework

**FIGURE 6:**
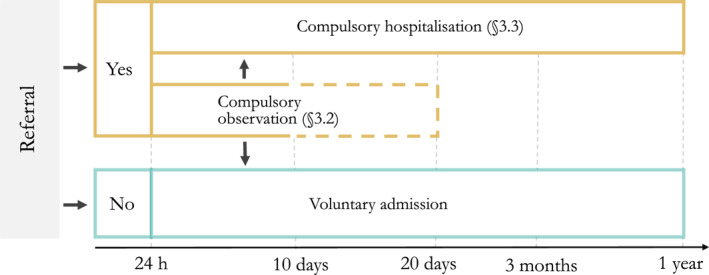
The Norwegian Mental Health Care Act regulates compulsory inpatient and outpatient mental health care. Decisions for compulsory care are made at referral; within 24 h of admission; within 10 days (for compulsory observation); at least every third month, and 1 year after referral for compulsory mental health care, but discharge can be decided at any time.

Individuals must be evaluated by a physician before being referred to inpatient compulsory mental health care. After arrival in hospital, a psychiatrist or specialist psychologist must re‐evaluate the patient within 24 h. Figure 6 shows Norway's legal framework for administrative compulsory mental health care.^2^ §3.2 governs compulsory observation, which can last up to 10 days and be extended 10 days §3.3 regulates compulsory inpatient and outpatient mental health care, which requires a severe mental illness, a treatment criterion (their prospects of recovery will be significantly reduced, or their condition will deteriorate significantly, without compulsory care) or danger criterion (the person constitutes a serious and immediate risk to the life or health of self or other). In cases not involving danger, the person must also lack decision‐making capacity. Medication without consent is only permitted for those in compulsory care and requires additional formal decisions.

## 5 DATA ACQUISITION AND MANAGEMENT

We will receive data from nine Norwegian registries, listed in Table A1 in the appendix. The Norwegian Patient Registry (NPR) will prepare and send a population list with personal identification numbers to Statistics Norway. They will draw a control group from The Central Population Register (CPR), matched on age and gender, which is identical to the treatment group, except that they were not referred to specialist mental health or addiction services. The CPR will send the list of personal identification numbers together with a project‐specific pseudonymised number back to the NPR and to the Institute of Public Health who manages the Cause of Death registry and the Norwegian Prescription Database. Statistics Norway will keep the linkage key between the pseudonymised number and the personal identification number for future data deliveries. The remaining registries' data will be attached by Statistics Norway and sent to Haukeland University Hospital, where it will be securely stored and analysed.

### 5.1 Study samples and target populations

Since the registers are mandatory, they typically contain data that is complete, reliable and representative. The project will obtain data on everyone above nine years who were referred to specialist mental health or addiction services in Norway 2015–2016. For WP1, we estimate that the patient sample and the equally sized control group from the general population will constitute approximately 600,000 individuals. For WP2 we will specify smaller target populations, with increased risk for compulsory mental health care. Relevant subgroups include people who have or will receive a diagnosis of SMI and people referred involuntarily during the study period.

The cohort period is limited to 2015–2016, but we will obtain data for both the cohort and control group for the period 2010–2025. This permits differentiation between the first episodes of SMI and patients with previous contact with specialist services.

The registry data will be supplemented with municipal data (See Table A1), which will be used to explore associations between structural and capacity‐related regional variables and rates of compulsory care and SMI. Furthermore, they can be used to adjust for different case‐mix in the catchment areas.

For both WPs it might be relevant to perform separate analyses for compulsory observation and compulsory mental health care, since the patient groups do not overlap completely and expected outcomes differ somewhat. Similarly, it can be relevant to perform subgroup analysis for some outcomes based on gender, diagnoses or other characteristics.

### 5.2 Assumptions of provider‐preference instrumental variables

First, the *relevance criterion* implies that the instrument must be correlated with the treatment variable. We intend to generate IVs at the regional provider level of catchment area to Community Mental Health Centres (Ruud and Friis, 2021). Stable geographical variations in compulsory hospitalisation between catchment areas have been documented, and more than 80% of variation in ranked rates of compulsory hospitalisation per population could be predicted by the area rankings for the previous year, while 50%–75% of variation in inpatient rates could be predicted this way (Hofstad et al., 2021), suggesting that provider preference for compulsory care can be a strong instrument (Hernán and Robins, 2020).

Second, the *exclusion restriction assumption* implies the instrument should only impact the outcome through treatment. This means that service providers' preferences for using compulsory mental health care in an area should impact whether involuntary care is initiated, while being uncorrelated with outcomes through other paths. We will use a *negative control population* to test this assumption (Lipsitch et al., 2010); since provider preference should have no impact on those who were not in contact with mental health services, the impact of the instrument on the outcome should be zero in the control cohort.

Theoretically, the instrument will control for unobserved and observed characteristics of individuals that can affect the outcome. However, instrument‐outcome confounders can still bias estimates, in which case the instrument would not be equivalent to “as‐good‐as‐random assignment” (Garabedian et al., 2014). So the third *assumption of no confounding* requires that there are no unmeasured common causes of the instrument and the outcome. This will be tested indirectly through balance tests of baseline covariates (Brookhart and Schneeweiss, 2007).

The fourth assumption of *monotonicity* ensures that the estimand can be interpreted as the Local Average Treatment Effect (LATE). Individuals who clearly fill criteria for compulsory care are assumed to be subject to compulsion in all areas, while persons where compulsory hospitalisation is clearly not indicated are not subject to compulsion anywhere. Consequently, the estimand represents the effect of compulsory care for people with SMI at the margin/in the grey zone of compulsory care; individuals who are expected to be treated differently if they live in areas with a high versus a low use of compulsory care (See Figure 2). By design, we know that all people who are assigned to the treatment (compulsory care), will receive it, thereby ensuring no ‘*defiers’*.

Subject‐matter knowledge, required for all but the first assumption, will be provided by project group members, as well as service‐users and providers. Topics meriting investigation include whether the variation is random or remains stable over time. Are there differences in case mix, with different prevalence of SMI between areas? Is there risk of selection bias, where service users residing in low‐compulsion areas, but require compulsory hospitalisation, are transferred to areas that use more compulsion?

In general, people are unaware whether rates of compulsory mental health care in their catchment area are high or low compared to the country average. Given the retrospective baseline, the quasi‐experimental framework is similar to a double‐blinded experiment where neither service users nor providers could identify the treatment. While some clinicians may have recognised that the use of compulsory care tended to be higher or lower within their area, they were unaware that they partook in a natural experiment. There is also little reason to expect that people self‐selected to areas based on compulsory hospitalisation rates, thereby reducing the risk of selection bias.

Even if the exclusion restriction assumption is violated, the ‘*reduced form’* can provide an unbiased estimate of the effect of the instrument on the outcome (Chernozhukov and Hansen, 2008), and will answer whether a higher or lower propensity for compulsory care results in superior, worse or similar outcomes for patients.

### 5.3 Ethical approval and consent

This project was approved by the Regional Ethical Committee for Medical and Health Services Research, Norway: REC South‐East, Committee D (REC number 2017/2436/REC south‐east D). The committee considered that the project had potential for considerable social utility, while preserving the participants' welfare and integrity. Furthermore, informed consent was considered infeasible to obtain, and criteria for exemption from the duty of confidentiality were fulfiled. Based on the projects' objectives and potential usefulness of results, the Committee approved that information from the different registries could be combined. A Data Protection Impact Assessment was performed and updated during the project development.

## 6 DISCUSSION

This protocol describes a novel approach to informing the debate about the effectiveness of compulsory mental health care, which can produce important new knowledge of societal interest and importance.

We envision three possible scenarios for each outcome, with different consequences for the ongoing debate; policy and legislation; and evidence‐based medicine. One scenario, which is not unlikely, is that we find no difference in long term outcomes for patients in areas with a higher or lower propensity for compulsory care usage. In this case, the burden of proof of effectiveness rests on the areas that frequently use compulsory care, given the many ethical challenges inherent in the practice.

A second scenario is that we find more positive long‐term outcomes in areas with more compulsory care. This scenario might put empirical data at odds with the ethical position that compulsion can rarely, if ever be justified, and can support compulsion use in some contexts. However, it will not invalidate many of the strong arguments for limiting compulsion, such as the inherent risk of harm and lack of respect for self‐determination.

The third scenario is more negative outcomes in areas with a higher propensity for using compulsion. This can indicate that benefits from compulsion are limited, with problematic secondary effects that harm services user/provider relations and impede compliance. Such a finding would provide more clear implications and strongly support continued efforts at reducing compulsion.

The observed geographical variation in compulsory mental health care in Norway reflects international trends. The findings from this project might also help inform the debate in countries that lack access to high‐quality registry data at a population level.

The extent to which the research community is detached from the controversy is questionable. While some variation in viewpoints exist, it appears the research community leans towards reducing coercion, as reflected in names such as FOSTREN: Fostering and Strengthening Approaches to Reducing Coercion in European Mental Health Services and ReCoN: Reducing Coercion in Norway. Our project group acknowledges the ethical challenges inherent in coercive mental health care (Chieze et al., 2021), but remains open to arguments from both sides of the controversy. Our group consists of individuals with differing opinions, ranging from those who believe compulsory care is rarely justified to those who believe it is often necessary. We aim to maintain a neutral position and prioritise empirical evidence in determining the best approach for patients. We are committed to publishing all findings, including null or unexpected results.

## AUTHOR CONTRIBUTION

TH wrote the first draft and created the figures. AM is PI for the project and responsible for ethics, funding, main supervision and data management. SM and DMD are involved for expertise on statistics, epidemiology, and IV‐methods. AB wrote the lived experience commentary. KR is head of the unit where the study belongs. TWH was involved in planning and applications. ON, EJ, EK, DMD, MR, Ki.D, BB, Ke.D, LG, SB, TP, MFS, AB, JR, BMH, RP and KR have advised on clinical; legal; ethical; and/or substance matter. All collaborators have contributed to the paper's intellectual content and have approved the final manuscript.

## CONFLICT OF INTEREST STATEMENT

The authors declare no conflicts of interest.

## ETHICS STATEMENT

Current ethics approvals last from 2022 to 2030. The project may be extended granted ethics approval and additional funding.

## Data Availability

Approvals from ethical committees and registry owners are limited to this project. Regulations and ethics approvals prohibit that the data be made public.

## ENDNOTES

^1^
We define compulsory care as formal compulsion through mental health legislation, meaning that other coercive practices such as seclusion and restraints are outside the scope of the project.

^2^
In addition, compulsory mental health care can be court‐ordered, as a special criminal sanction, cf. Penal code Section 62, but such forensic compulsory care is not included in this study.

## Appendix

**TABLE A1:** Data sources for the project, with examples of available variables.

National register name	Variables
Norwegian patient register (NPR)	ICD‐10 diagnosis code (F‐diagnoses); institution; in/out date; referral status; judicial reason for stay; involuntary admission;Outpatient commitment; profession deciding legal status
The central population register (CPR)	Gender; age; in/out migration; categories of immigration background; current municipality
The central penal and police register (SSP)	Charges for nine crime groups: 1. Property theft; 2. Other offences for profit; 3. Criminal damage; 4. Violence and maltreatment; 5. Sexual offences; 6. Drug and alcohol offences; 7. Public order and integrity violations; 8. Traffic offences; 9. Other offences); date of crime; prison; victim of crime
Norwegian cause of death registry	Cause of death; month
Norwegian education database (NUDB)	Highest completed level of education; mother's/father's educational level;Ended high school education before completion (“dropout”)
Tax, income and welfare registry	Income; work assessment allowance; social assistance; disability pension; unemployment
Norwegian prescription database (NorPD)	Medications for psychiatric disorder (chapter N in the ATC system); defined 24 hours dose (DDD); prescription date (quarter)
Municipal patient and user register (KPR)	Type of service; type of function; private help; type of residence; extent of service (hours pr. week);Refused service; municipality
Control and health reimbursement register (KUHR)	Types of injuries/problems (somatic and psychiatric); for treatment in first line emergency clinics
Municipal data from statistics Norway	Proportion of high school dropouts; median income; bed capacity in mental health care
Municipal data from Norwegian labour and welfare administration	Unemployment rate
